# Complete pathologic response of HER2-positive breast cancer liver metastasis with dual Anti-HER2 antagonism

**DOI:** 10.1186/1471-2407-14-242

**Published:** 2014-04-04

**Authors:** Hans F Schoellhammer, Felicia Hsu, Courtney Vito, Peiguo Chu, Jinha Park, James Waisman, Joseph Kim

**Affiliations:** 1 Division of Surgical Oncology, Department of Surgery, City of Hope Comprehensive Cancer Center, MD, 1500 E. Duarte Rd., Duarte, CA 91010, USA; 2 Department of Pathology, City of Hope Comprehensive Cancer Center, 1500 E. Duarte Rd., Duarte, CA 91010, USA; 3 Department of Radiology, City of Hope Comprehensive Cancer Center, 1500 E. Duarte Rd., Duarte, CA 91010, USA; 4 Department of Medical Oncology and Experimental Therapeutics Research, City of Hope Comprehensive Cancer Center, 1500 E. Duarte Rd., Duarte, CA 91010, USA

**Keywords:** HER2-positive breast cancer, Targeted therapy, Breast cancer liver metastases, Trastuzumab, Pertuzumab, Complete pathologic response

## Abstract

**Background:**

Although breast cancer frequently metastasizes to the bones and brain, rarely breast cancer patients may develop isolated liver metastasis. There is increasing data that anti-HER2 targeted therapy in conjunction with systemic chemotherapy may lead to increased rates of pathologic complete response in the primary breast cancer. However, little is known about its effects on metastatic liver disease.

**Case presentation:**

We report the treatment of a 54-year-old female who was diagnosed with HER2-positive invasive ductal carcinoma and synchronous breast cancer liver metastasis (BCLM). The patient underwent eight cycles of standard docetaxel with two anti-HER2 targeted agents, trastuzumab and pertuzumab. Subsequent radiographic imaging demonstrated complete radiographic response in the primary lesion with an approximate 75% decrease in the liver metastasis. After informed consent the patient underwent modified radical mastectomy that revealed pathologic complete response. Re-staging demonstrated no new disease outside the liver and a left hepatectomy was performed for resection of BCLM. Final pathologic examination revealed no residual malignant cells in the liver specimen, indicating pathologic complete response. Herein, we discuss the anti-HER2 targeted agents trastuzumab and pertuzumab and review the data on dual HER2 antagonism for HER2-positive breast cancer and the role of surgical resection of BCLM.

**Conclusions:**

The role of targeted agents for metastatic HER2-positive breast cancer is under active clinical trial investigation and we await the maturation of trial results and long-term survival data. Our results suggest that these agents may also be effective for producing considerable pathologic response in patients with BCLM.

## Background

Breast cancer is a major public health concern and affects tens of thousands of women worldwide each year. In approximately 25% of patients, the breast cancer cells over-express human epidermal growth factor receptor-2 (HER2) on the cell surface, which results in a more aggressive breast cancer phenotype and significantly decreased overall and disease-specific survival compared with patients whose breast cancer does not overexpress HER2 [1]. Monoclonal antibodies, such as trastuzumab, that bind to HER2 proteins can be used along with chemotherapy to treat patients with HER2-overexpressing breast cancer with metastases to organs outside of the breast. In this paper we present a case of HER2-positive breast cancer liver metastasis successfully treated with anti-HER2 targeted therapy resulting in a complete pathologic response.

## Case presentation

**Figure 1:**
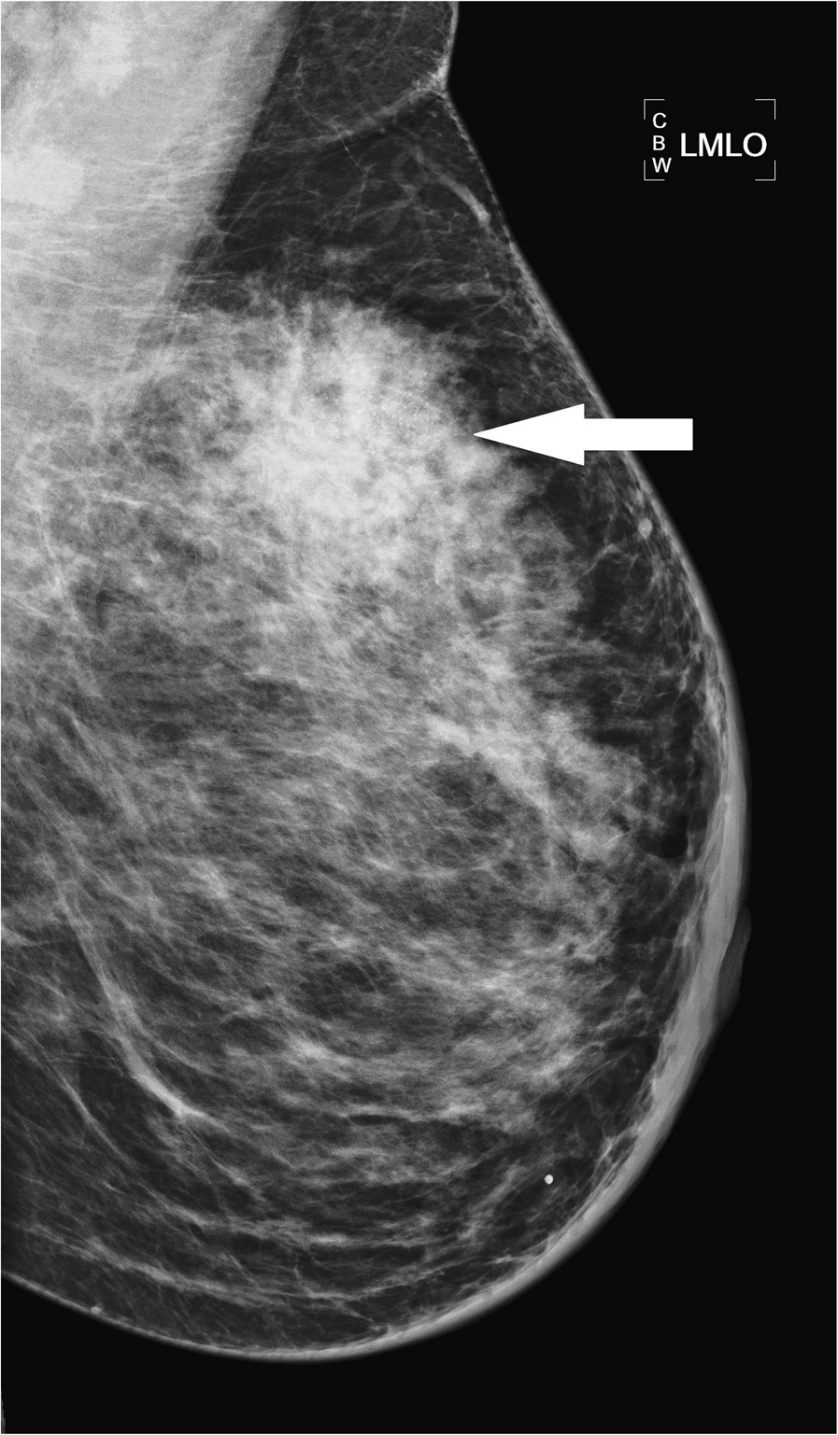
Medial-lateral oblique mammogram of the left breast demonstrating a large spiculated mass with calcifications in the upper aspect of the breast (marked by arrows); biopsy of the mass revealed HER2-overexpressing infiltrating ductal breast cancer.

**Figure 2:**
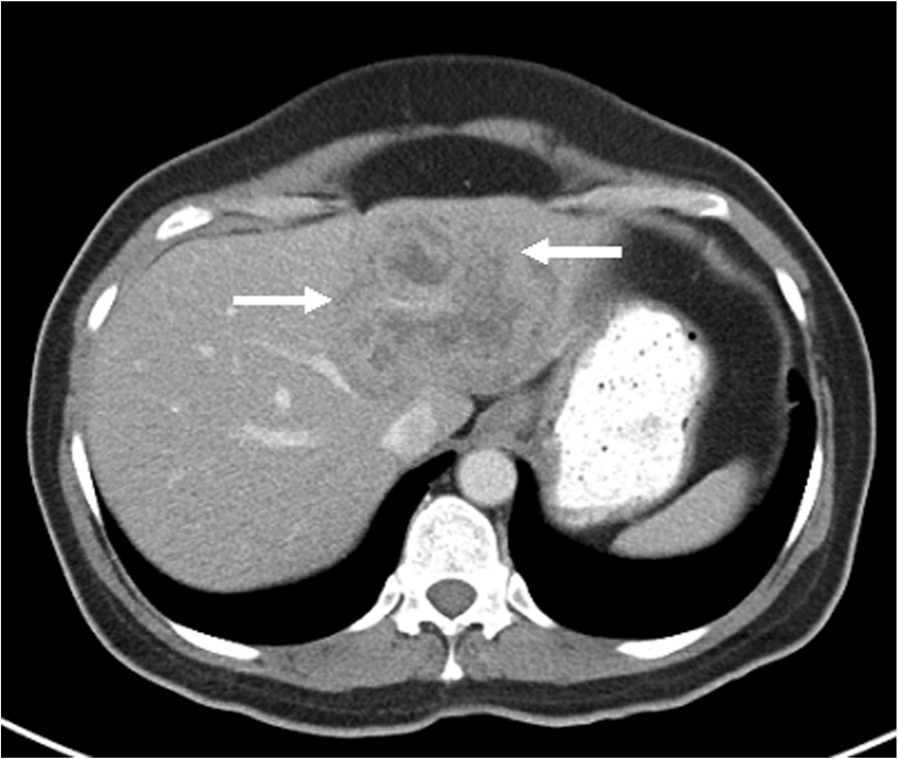
Pre-treatment CT scan of the abdomen showing a large hypodense mass in the left lobe of the liver (marked by arrows); biopsy of the mass revealed metastatic HER2-positive breast cancer.

**Figure 3:**
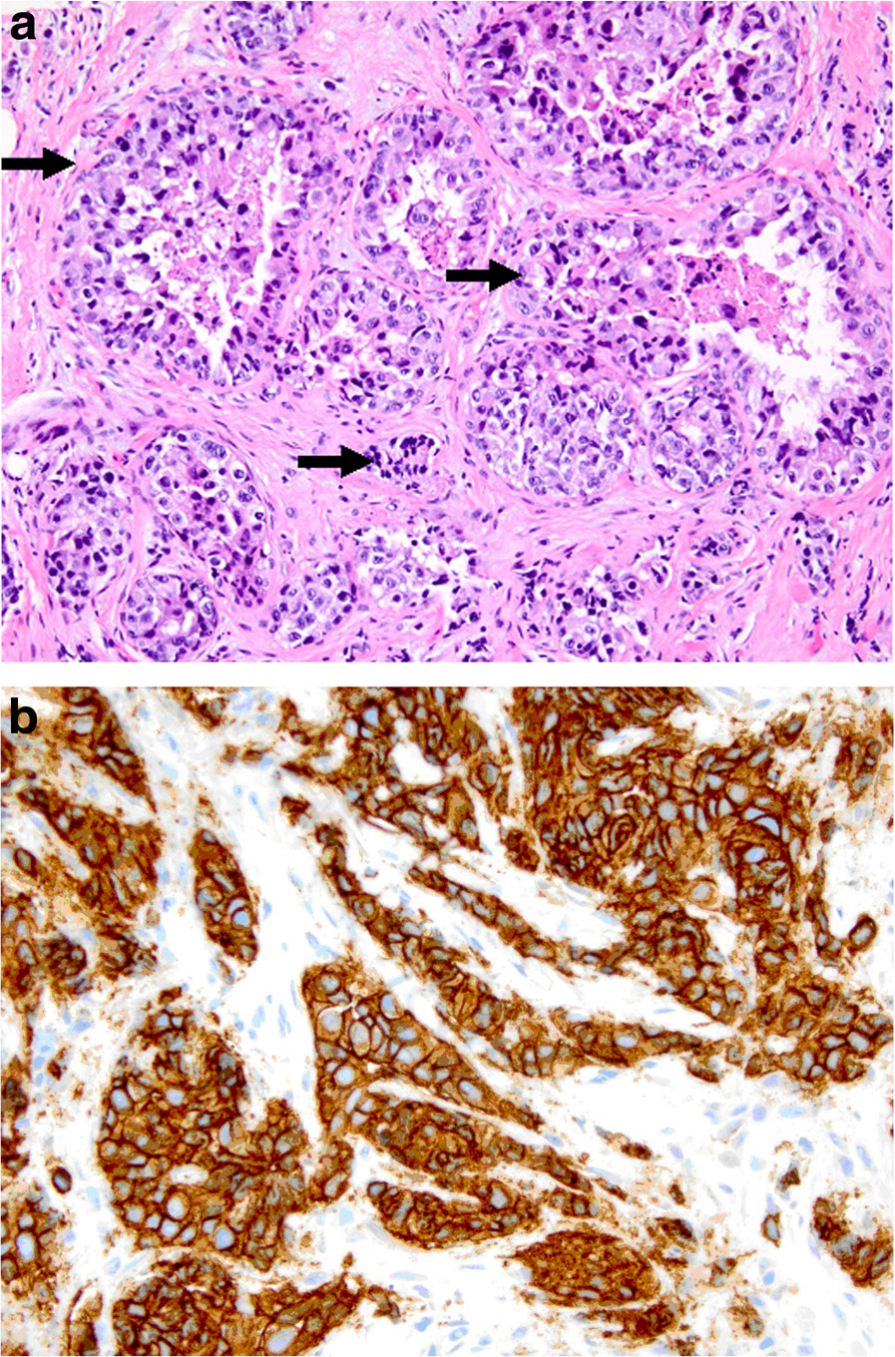
**Photomicrographs of the primary left breast infiltrating ductal carcinoma.** Figure 3**a** demonstrates carcinoma cells (marked with arrows) stained with hematoxylin and eosin (200X magnification). Figure 3**b** demonstrates intense 3+ breast cancer cell surface staining on immunohistochemistry indicating HER2 overexpression (400X magnification).

A 54-year-old Caucasian female with no past medical history or co-morbidities presented to an outside institution with 3-month history of an enlarging palpable mass in her left breast associated with skin thickening and nipple retraction. The patient reported rapid growth of the mass over the preceding month. Mammography was ordered and revealed a 10 × 4 × 6 cm mass in the upper outer quadrant of the left breast associated with pleomorphic calcifications (Figure 1). Ultrasound-guided biopsy of this ill-defined hypoechoic mass demonstrated poorly-differentiated, grade 3 of 3, ER-negative, PR-negative, HER2-positive infiltrating ductal carcinoma. Biopsy of an enlarged 1.4 cm left axillary lymph node revealed metastatic adenocarcinoma. Human epidermal growth factor receptor-2 (HER2) protein expression was 3+ by immunohistochemistry and HER2 gene was amplified with a ratio of 6.7 by fluorescence in situ hybridization; Ki-67 was markedly elevated at 50%. High-grade comedo and solid ductal carcinoma in situ (DCIS) was also identified. Metastatic workup with computed tomographic scans of the chest, abdomen, and pelvis revealed an 8.2 × 6.8 cm mass in the left lobe of the liver (Figure 2), but no evidence of metastatic disease elsewhere. The liver lesion was biopsied and showed adenocarcinoma that was ER/PR-negative and HER2-positive (Figure 3a and 3b), consistent with metastatic breast cancer.

Given the HER2-positive status, the patient was scheduled to receive chemotherapy in combination with HER2-targeted monoclonal antibody trastuzumab, which binds to HER2 and disrupts cell signaling and proliferation [1]. Prior to the initiation of therapy, the US Food and Drug Administration approved another anti-HER2 targeted monoclonal antibody, pertuzumab, for first-line treatment of HER2-positive metastatic breast cancer in combination with docetaxel and trastuzumab. The approval was based on results from the randomized Phase III Clinical Evaluation of Pertuzumab and Trastuzumab (CLEOPATRA) trial which showed increased progression-free survival (PFS) in HER2-positive metastatic breast cancer patients treated with docetaxel, trastuzumab, and pertuzumab compared to docetaxel and trastuzumab alone.

The patient underwent eight cycles of docetaxel (75 mg/m^2^ every three weeks), trastuzumab (8 mg/kg loading dose on Day 2 of the first cycle followed by 6 mg/kg every three weeks thereafter), and pertuzumab (840 mg loading dose on Day 2 of the first cycle followed by 420 mg every three weeks thereafter) over a total period of six months. The patient tolerated therapy without adverse effects and underwent re-staging with PET/CT after the 4th cycle of treatment, demonstrating near 75% reduction in the breast lesion. Additionally, the liver metastasis decreased in size from 8 cm to 5 cm. Re-staging imaging studies after the 8th cycle of therapy showed radiographic resolution of the left breast mass and interval decrease of the liver mass to 2 cm.

Since retrospective studies have suggested improved survival for patients with stage IV breast cancer with resection of the primary tumor [2,3] and given the patient’s remarkable therapeutic response, consideration was given to resection of the primary breast cancer following the 8th treatment cycle. As such, the patient underwent left modified radical mastectomy with tissue-expander reconstruction seven months after the diagnosis of stage IV breast cancer was made. Final pathologic examination revealed residual high-grade DCIS with necrosis (2.7 cm); however no residual invasive carcinoma was identified. Therefore, pathologic complete response (i.e., ypTisN0M1) of the invasive tumor was observed. Due to the original size of the primary lesion, the patient received standard post-mastectomy radiation therapy to the left chest wall and nodal basin. After the breast operation the patient was continued on trastuzumab 6 mg/kg and pertuzumab 420 mg given every three weeks.

**Figure 4:**
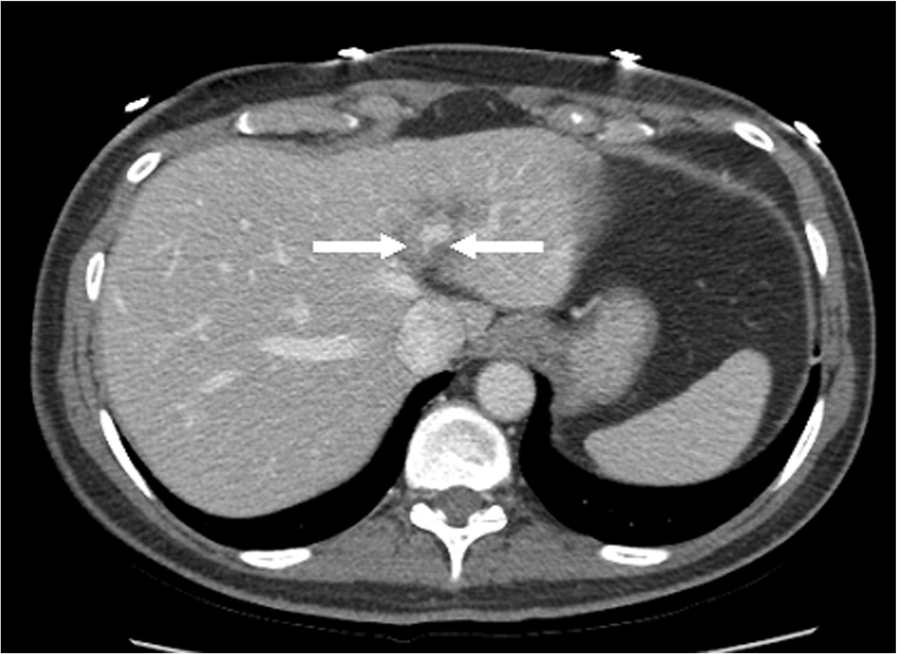
CT scan of the abdomen showing a dramatic decrease in size of the metastasis (marked by arrows) in the left lobe of the liver after treatment with eight cycles of pertuzumab, trastuzumab, and docetaxel.

**Figure 5:**
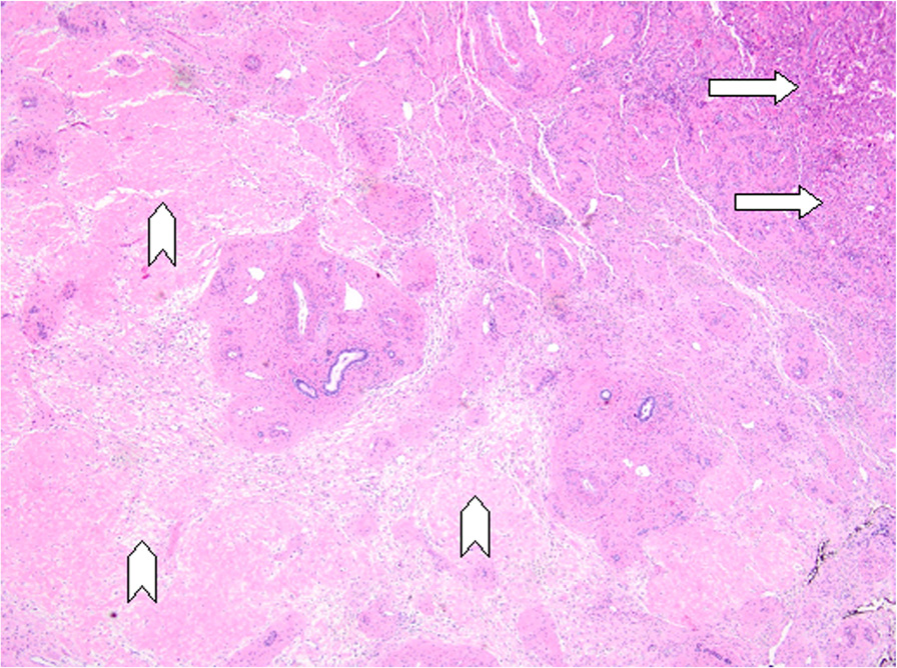
Photomicrograph of the left hepatectomy specimen stained with hematoxylin and eosin, demonstrating normal liver parenchyma marked with arrows, fibrotic tissue with hyalinization and scattered lymphocytic infiltrate (marked by arrowheads) without evidence of breast cancer cells, consistent with response to treatment and indicating complete pathologic response (40X magnification).

**Figure 6:**
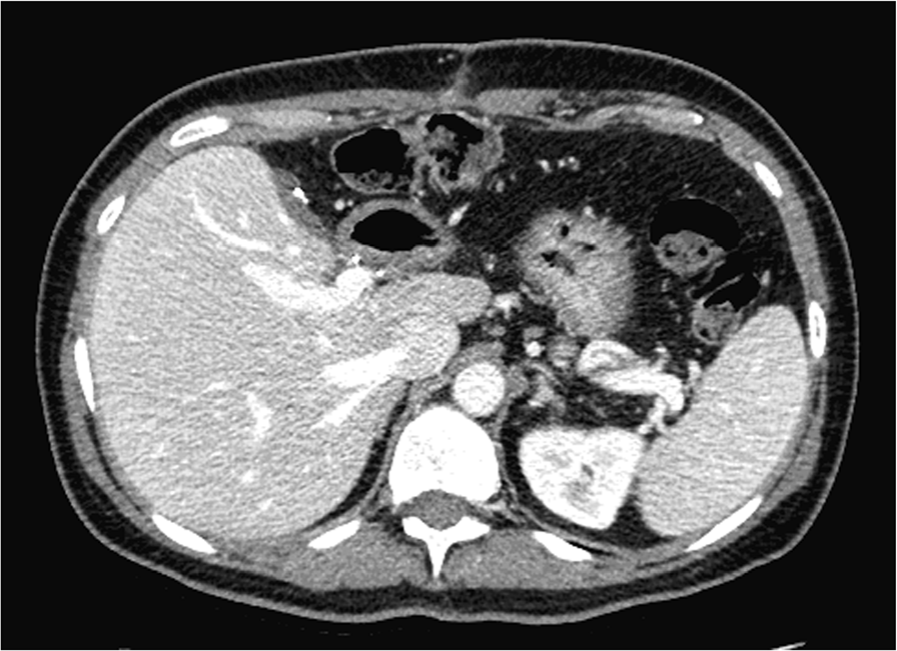
CT scan of the abdomen performed three months after resection of the left lobe of the liver demonstrating normal-appearing right lobe of the liver without evidence of new or recurrent metastatic disease.

The patient subsequently transferred her care to our institution and was evaluated for resection of the liver metastasis. Triple-phase CT scan at our institution taken 12 months after her initial presentation revealed the left hepatic lobe metastasis to be 2.3 × 2 cm without evidence of metastatic disease elsewhere (Figure 4). The liver lesion was deemed to be resectable and the patient underwent left hepatectomy approximately five months after modified radical mastectomy had been performed. The patient’s post-operative course was uncomplicated and she was subsequently discharged home in excellent condition. Final pathologic examination of the resected specimen revealed an area of scar tissue with stromal hyalinization, scattered histiocytes, and lymphocytic infiltrate measuring 1.2 cm. No residual malignant cells were identified in the resected liver, thus indicating a complete pathologic response (Figure 5). On surveillance imaging approximately three months after resection, repeat CT of the abdomen/pelvis demonstrated no evidence of new or recurrent disease in the liver (Figure 6). The patient continues to do well without disease approximately 6 months after liver surgery. Currently the optimal duration of anti-HER2 therapy for patients with long-term disease control is not known [4], and as such the patient will remain on dual agent pertuzumab and trastuzumab given every three weeks indefinitely.

## Discussion

Breast cancer is the most common cancer in women worldwide, accounting for 1.3 million new cases in 2008 (23% of all new cases) [5]. Ten to 15% of patients have metastatic disease at the time of initial presentation [6], and the most common sites of metastases are the bones and brain with only 1-5% of breast cancer patients developing isolated liver metastasis [7,8]. Aggressive tumor biology and corresponding poorer prognosis is associated with amplification or overexpression of HER2, a transmembrane tyrosine kinase protein belonging to the human epidermal growth factor receptor (EGFR) family of proteins [9]. Historically, patients with HER2-positive breast cancer have had poor prognosis, with response rates to chemotherapy ranging from 17-42% [1]. Now with the advent of anti-HER2 therapy, tumor response and patient survival have dramatically improved [10,11].

The first anti-HER2 targeted agent was the monoclonal antibody trastuzumab, which initially was approved for the treatment of HER2-overexpressing breast cancer with standard chemotherapy in the metastatic setting. Trastuzumab inhibits ligand-independent HER2 activity and related downstream signaling by binding to its extracellular domain [12,13]; however trastuzumab binding does not interfere with HER2 heterodimerization, which mediates downstream cell proliferation [14]. Pertuzumab, the second commercially approved selective anti-HER2 agent, may act in synergy with trastuzumab to antagonize HER2 signaling by blocking HER2 heterodimerization and activating antibody-dependent cell-mediated cytotoxicity [15].

Treatment of HER2-positive breast cancer patients with dual anti-HER2 antagonism translates to better therapeutic responses. In the CLEOPATRA trial approximately 80% of patients randomized to the experimental treatment (docetaxel, trastuzumab, and pertuzumab) had an objective tumor response compared to 69.3% with control treatment (docetaxel and trastuzumab) [13]. In a Phase II trial by Baselga *et al.*, patients with metastatic HER2-positive breast cancer received trastuzumab with pertuzumab and had response rates of 24.2%, and 7.6% of patients had a pathologic complete response [16]. In another Phase II trial, the Neoadjuvant Study of Pertuzumab and Herceptin in an Early Regimen Evaluation (NeoSphere) Trial, the highest rates of pathologic complete response were observed in patients receiving docetaxel, trastuzumab, and pertuzumab. Interestingly, in patients receiving targeted therapy alone (i.e., trastuzumab and pertuzumab) approximately 17% of patients had pathologic complete response, demonstrating that dual HER2 inhibition alone may elicit remarkable responses in HER2-positive breast cancers [17]. Unfortunately, none of the aforementioned trials specifically characterize metastatic liver disease and it is unclear whether such results could reasonably be applied to any metastatic site.

Our experience indicates that HER2-overexpressing BCLM can be effectively treated with chemotherapy and dual HER2 targeted therapy. This is important for patients with isolated liver metastases (1-5% of all metastatic patients), because control and possibly cure of the disease can be achieved. Indeed, liver resection has become a treatment option for selected patients with BCLM. Prior to the modern era, older studies showed no survival advantage for metastatic breast cancer patients who underwent liver resection, with five-year survival of 9% seen [18-20]. Now, contemporary studies routinely report survival advantages in select patients undergoing liver resection for BLCM. Five-year overall survival rates approaching 21%-38% are the norm with a combination of chemotherapy and resection, and a wide variety of chemotherapeutic regimens have been reported to be used in the literature, commonly Adriamycin/cyclophosphamide or cyclophosphamide/methotrexate/fluorouracil [7,8,21]. The survival rate of breast cancer patients with isolated liver metastasis who have undergone liver resection has dramatically increased due to medical advances and multidisciplinary care: improved chemotherapy and targeted agents, more effective surgery and better post-operative care. For patients with HER2-positive BCLM, we expect that outcomes in the future will be even further improved given the high rates of tumor response to HER2 targeted therapy and the possibility of achieving a complete pathologic response.

## Conclusion

The role of HER2 targeted agents such as pertuzumab will continue to evolve in the treatment of patients with BCLM, and may lead to curative therapeutic plans. There is no data on pathologic complete response from this new treatment option and we anticipate that our experience may prove in the future to be a common and frequent outcome. Targeted agents in combination with chemotherapy will undoubtedly increase the resectability of liver metastasis. Continued multi-disciplinary treatment strategies will be essential in the future to coordinate the roles of targeted therapy and liver resection, ultimately with the goal of providing patients improved survival benefit.

### Consent

Written informed consent was obtained from the patient for publication of this Case Report and any accompanying images. A copy of the written consent is available for review by the Editor of this journal.

## Competing interests

The authors declare that they have no competing interests.

## Authors’ contributions

HFS obtained the radiographic and pathologic images and drafted the manuscript. FH and CV helped to draft the manuscript. PC read the pathologic slides, captured the images, and helped draft the manuscript. JP read the radiographic images and helped draft the manuscript. JW helped draft the manuscript. JK conceived of the case report, participated in its design and coordination, and helped draft the manuscript. All authors read and approved the final manuscript.

## Pre-publication history

The pre-publication history for this paper can be accessed here:

http://www.biomedcentral.com/1471-2407/14/242/prepub
